# A solvent-free microbial-activated air cathode battery paper platform made with pencil-traced graphite electrodes

**DOI:** 10.1038/srep28588

**Published:** 2016-06-23

**Authors:** Seung Ho Lee, Ju Yeon Ban, Chung-Hun Oh, Hun-Kuk Park, Samjin Choi

**Affiliations:** 1 Department of Medical Engineering, Graduate School, Kyung Hee University, Seoul 02447, Korea; 2 Department of Medical Laser, Graduate School, Dankook University, Cheonan 31116, Korea; 3 Department of Biomedical Engineering, College of Medicine, Kyung Hee University, Seoul 02447, Korea

## Abstract

We present the fabrication of an ultra-low cost, disposable, solvent-free air cathode all-paper microbial fuel cell (MFC) that does not utilize any chemical treatments. The anode and cathode were fabricated by depositing graphite particles by drawing them on paper with a pencil (four strokes). Hydrophobic parchment paper was used as a proton exchange membrane (PEM) to allow only H^+^ to pass. Air cathode MFC technology, where O_2_ was used as an electron acceptor, was implemented on the paper platform. The bioelectric current was generated by an electrochemical process involving the redox couple of microbial-activated extracellular electron transferred electrons, PEM-passed H^+^, and O_2_ in the cathode. A fully micro-integrated pencil-traced MFC showed a fast start-time, producing current within 10 s after injection of bacterial cells. A single miniaturized all-paper air cathode MFC generated a maximum potential of 300 mV and a maximum current of 11 μA during 100 min after a single injection of *Shewanella oneidensis*. The micro-fabricated solvent-free air cathode all-paper MFC generated a power of 2,270 nW (5.68 mW/m^2^). The proposed solvent-free air cathode paper-based MFC device could be used for environmentally-friendly energy storage as well as in single-use medical power supplies that use organic matter.

Small, light-weight, and portable power source devices have attracted widespread attention in recent years due to the rapid increase in energy demand^1^. Biofuel cells, which are bioelectrochemical devices that convert biochemical energy into electrical energy, are regarded as a renewable energy source technology due to the high turnover rates related with the enzymes or microorganisms that they use as catalysts^2^. Most research associated with renewable fuel sources have adopted enzyme-based biofuel cells (EBCs) including ionic liquid-functionalized nanotubes, enzymatic biocatalysts, enzyme-functionalized graphene nanosheets, and various carbon electrodes^1^,^2^,^3^,^4^,^5^,^6^,^7^,^8^. Although EBCs produce high power densities, they encounter many problems including those related to the high cost of enzymes^9^ as well as limited lifetimes due to the temperature, humidity, pH, and toxic chemicals^10^,^11^. Alternatively, because microbial fuel cells (MFCs) employ abundant, non-toxic, and relatively inexpensive organic matter, they produce clean and sustainable energy. However, they have lower power densities compared to EBCs^11^,^12^,^13^,^14^,^15^,^16^.

MFCs typically consist of anodic and cathodic chambers separated by a proton exchange membrane (PEM) that permits only H^+^ or other cations to pass from the anode to the cathode. Inoculated bacterial cells oxidize the organic matter that is present in the anolyte, generate electrons through microbial metabolism, and extracellularly release these electrons to the anode. The captured electrons are delivered through an external conductive load to the cathode to reduce an oxidant. This electrochemical process produces the current in MFC devices^15^,^17^,^18^,^19^,^20^,^21^,^22^. In order to maintain charge neutrality, protons in the anolyte will diffuse through the PEM to the cathode chamber simultaneously with electron transfer^15^,^17^. The MFC performance can be evaluated by analyzing the current density, maximal output power density, and sustainability^15^. However, conventional MFCs are very expensive large-scale devices with long start-up times and considerable chamber volumes. These limitations have stimulated the fabrication of milliliter- or microliter-scale MFCs^14^,^19^. Miniaturized MFCs provide unique features including short electrode distances, large surface areas, fast response times, and low Reynolds numbers.

Paper-based microfluidic analytical devices have been functionalized as platforms for performing rapid diagnostic tests that require biochemical sensing^23^. In recent years, paper-based disposable devices have begun to be employed as battery devices;^24^,^25^ however, conventional batteries are not suitable for single use due to their high cost and toxicity. These limitations have led to an increased demand for low-cost, nontoxic, portable power sources. Paper, which is inexpensive, easily available, disposable, thin, lightweight, biocompatible, and biodegradable^26^,^27^, is considered to be an alternative electrode material for battery devices. The most attractive feature of paper is its momentary fluid suction, through capillary action, that does not require an external power source. Some studies have introduced paper-based bacterial metabolism-catalyzed battery platforms^2^,^8^,^12^,^18^,^28^,^29^,^30^,^31^. However, most of this research has produced higher electrical power through additional chemical treatments on the paper, including electron-catching printing materials (electrode), ferricyanide (electron acceptor), and Nafion solutions (PEM). Despite the advantages of Nafion, it is not suitable for application in a resource-limited setting due to its toxicity and expense. Therefore, we introduce a solvent-free microbial-activated air cathode battery paper platform (Fig. 1), using pencil-traced graphite electrodes, as a portable power source. Our extremely low-cost, miniaturized, all-paper microbial electricity-generating device requires no additional chemical treatments. Graphite-based anodes deposited by a pencil on paper were used to enhance bacterial adhesion. The ambient air on the pencil-traced cathode was used an electron acceptor (i.e., an air cathode)^11^,^12^,^14^,^15^,^16^,^30^,^32^,^33^. Parchment paper, purchased from a bakery, was used to pass only H^+^ from the anode to the cathode. Two hollow channels with wax-printed boundaries were adopted for sustainable electrical generation. To the best of the authors’ knowledge, although two studies^30^,^34^ previously reported no additional chemical-treated paper PEMs such as the bare paper and parchment paper, they showed paper-based 48-well MFC array using customized PCB boards and/or no properties of graphite-based electrode. Therefore, this study improved the performance of bioelectricity generation by combining several advantages of previous studies.

## Results and Discussion

### Potential of the pencil-traced electrode on paper

This study involves the fabrication of a bacterial metabolism-catalyzed battery platform by depositing graphite particles onto the fibers of cellulose. The most attractive point of this approach is the facile fabrication of the anode and cathode, which is done by drawing them on paper with a pencil (Fig. 2A). Drawing on paper is an old, well-established daily action, requiring only a paper and a pencil or ink^35^. Paper consists of cellulose fibers with a 3D hierarchical arrangement showing a rough and porous structure^28^. Pencil lead consists of graphite particles and clay. The bare paper (Fig. 2B) showed an irregular arrangement of cellulose fibers in a 3D network, while the 8B pencil trace on paper (Fig. 2C) showed a variety of densely and irregularly deposited graphite flakes on the fibers of cellulose or between cellulose fibers. The pencil-traced paper contained interconnected graphitic layers with many edges and boundaries. The Raman spectra of the pencil trace showed prominent Raman peaks at around 1320 and 1590 cm^–1^; these can be assigned to the D and G bands of carbon materials, respectively. The D band corresponds to the presence of defects and disorder in the form of edges and grain boundaries, and the G band corresponds to stretching of the sp^2^-bonded carbon lattice. Raman peaks corresponding to the cellulose signatures^36^ can be clearly observed (Table S1). The higher I_D_/I_G_ ratio of the pencil-traced paper is responsible for the lower structural alignment^37^. The rigid materials used in most MFC batteries result in slow electrical production after bacterial injection, which typically takes several days to a week^11^,^19^,^31^,^38^. However, paper-fabricated chambers or reservoirs allow for immediate electrical production as soon as the bacterial injection occurs due to the enhanced bacterial adhesion^11^,^18^,^29^,^34^. Rapid adsorption of the bacteria-containing liquid was due to the naturally rough and porous structure of paper. Although the pencil-traced paper showed poor structural alignment, it may be effective for holding media and enhancing bacterial adhesion, which can lead to an increase in the power production. Therefore, pencil-traced electrodes on paper are likely to be facile, low-cost, and innovative battery platforms that can be used as power sources for single-use diagnostic devices.

### Selection of pencil hardness

The properties of conducting electrodes are critical for evaluating the performance of battery platforms. They can determine the ratio of graphite that is deposited on the pencil-traced paper. Pencils are typically classified from 9H to 9B depending on their ratio of graphite and clay. An H-grade indicates a higher clay content (hardness), while a B-grade indicates a higher graphite content (blackness)^36^. HB pencils typically contain 60–70% graphite and 30–40% clay binder^39^. The morphological, molecular, and electrochemical properties of the pencil traces for four pencil hardnesses (i.e., 8B, 4B, HB, and 4H, as shown in Fig. S4) and a commercially-available flexible graphite sheet (control group) were evaluated in this study.

The softest 8B pencil produced the darkest-colored trace, while the hardest 4H pencil produced the lightest trace due to the different concentrations of graphite particles (Fig. S5). The pencil traces on paper (four strokes) deposited large amount of graphite as well as various graphite flakes, depending on the pencil hardness (Fig. 3), while the graphite sheet showed a smooth surface with few defects (Fig. S6). In particular, the 8B pencil trace deposited the largest amount of graphite on the paper, while the 4H pencil trace deposited no graphite components on the paper. EDX spectra (Fig. 4A) confirmed the presence of C, O, and Si in all of the substrates, regardless of pencil hardness. The graphite sheet showed the highest proportion of carbon material (99.8% purity); this was followed by the 8B, 4B, HB, and 4H pencil traces. Softer pencils (8B pencil) contained a higher relative proportion of carbon material, while harder pencils (4H pencil) contained a higher relative proportion of oxygen. This finding was consistent with the results of Lin *et al*.^40^. Since the electrical properties of electrodes depend on the amount of carbon materials, the 8B pencil was the best selection (in terms of both the morphological and molecular properties) for the fabrication of pencil-traced conducting electrodes.

The electrical conductivities of the graphite sheet and the 8B, 4B, HB, and 4H pencil-traced papers were determined to be 3716.3 ± 507.8, 75.8 ± 7.3, 284.0 ± 82.6, 0.0 ± 0.0, and 0.0 ± 0.0 μS/m, respectively (Fig. 4B). The graphite sheet (control) showed the highest electrical conductivity. This was followed by the 4B, 8B, and HB/4H pencil traces. The 4B pencil-traced paper showed higher conductivity compared to the 8H pencil-traced paper due to the different thermal treatments used while manufacturing the pencils. The highest conductivity of the graphite sheet confirmed the high degree of intralayer condensation of graphite^37^. Since the power density depends on the electrical conductivity of the electrodes, the 4B pencil was the best pencil for fabrication of pencil-traced conducting electrodes. The next best pencil was the 8B pencil. Additionally, we confirmed that the electrical conductivity of the 6B pencil-traced paper was higher than that of the 4B pencil-traced paper (not shown). Veerubhotla *et al*.^30^ reported on a paper-based instant power generation platform that used a 6B pencil-based bacterial biofuel cell; however, they did not mention why they selected the 6B pencil.

The Raman spectra of the graphite sheet and four pencil-traced papers showed prominent Raman peaks at around 1320 and 1590 cm^−1^. These are assigned to the D (defects and disorder) and G (graphitic) bands of carbon materials, respectively (Figs 4C and S7). The estimated I_D_/I_G_ ratios of each electrode were 0.25 (graphite sheet, Fig. S7), 3.71 (8B pencil), 3.21 (4B pencil), 3.13 (HB pencil), and 3.23 (4H pencil). The lower D/G intensity ratio of the graphite sheet indicated a more perfect graphitic structure, while the higher D/G intensity ratio of the pencil-activated graphite electrodes indicated a poorer graphitic structure^37^. The crystallite size of graphitic domains (*L*_*a*_) can be calculated using the laser line wavelength (*λ*_laser_) and the D/G intensity ratio, as follows:^41^,^42^


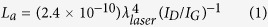


Since our diode laser source (*λ*_laser_) was 785 nm, the average crystallite sizes of the graphitic domains for each electrode were 364.5 nm (graphite sheet), 24.6 nm (8B pencil), 28.4 nm (4B pencil), 29.1 nm (HB pencil), and 28.2 nm (4H pencil). These results indicated that the pencil-traced electrodes have a poor structural alignment; however, since they consist of small graphite particles (relative to the size of the graphite sheet with good graphitic structure), the paper-based nano-graphitic pencil-traced electrodes have large surface areas and may yield superior electrical power production compared to graphite sheet electrodes, particularly for the 8B pencil (the best selection).

### Reproducibility of the pencil-traced electrode

Since the fabrication of the pencil-traced paper has a significant effect on battery performance, its reproducibility was evaluated according to the pencil hardness. Figure 4D shows representative pencil-traced hydrophilic bacteria-active areas (with four strokes) on paper according to the pencil hardness. Since the efficiency of the pencil-traced electrode is mainly decided by the physical properties of the paper-based substrate (i.e., the surface roughness)^43^, the material properties of the pencil lead^40^, and the writing force, two force conditions were evaluated: “normal” tracing (①–③, 8.34 ± 0.79 N) and “pressing-down” tracing (④ and ⑤, 15.78 ± 1.38 N). The mean RGB intensities of the 8B, 4B, HB, and 4H pencil-traced papers were 16.3, 103.3, 181.5, and 198.5, respectively. The 8B pencil-traced paper showed the highest relative standard deviation (RSD) of 4%. This was followed by HB (RSD = 9%), 4H (RSD = 10%), and 4B (RSD = 19%). This is similar to the pattern observed for the electrical conductivity (Fig. 4B). This finding demonstrates that the RGB intensity of the 8B pencil-traced electrode is close to being black in color and that the RGB intensities of the HB and 4H pencil-traced electrodes are close to being white in color. Alternatively, the RGB intensity of the 4B pencil-traced electrode is located on the boundary between black and white. Interestingly, using a different writing force with the pencil did not critically affect the fabrication reproducibility. However, in the future, we plan to achieve higher reproducibility during electrode fabrication by using an XY-plotter robot kit (Fig. S8). Since a blacker color indicates a higher graphite content, the 8B pencil with the higher electrode reproducibility (4%) was indirectly determined to be the best pencil for fabricating pencil-traced conducting electrodes.

### Fabrication of air cathode paper-based battery

From the results regarding the electrical properties (carbon content), electrical conductivity, nano-graphite surface roughness, and electrode reproducibility, the 8B pencil and graphite sheet were selected as the best electrode materials for paper-based air cathode batteries. A hydrophobic inlet layer was used to prevent leakage of the bacteria-containing liquid and air penetration^30^. A two-layered hollow chamber^44^,^45^,^46^ was selectively adopted for the battery in order to continuously supply bacteria in the hydrophobic-bounded single anode. The inoculated bacteria-containing fluid passes through the anode and the anodic chamber before reaching the PEM layer. To reduce the device cost and size, commercially-available hydrophobic parchment paper (<50-μm-thick) was used as a PEM^11^,^29^,^34^. This can prevent the bacteria in the anode from reaching the cathode. Veerubhotla *et al*.^30^ used a two-layered hydrophilic bare paper (Whatman filter paper) as a separator between the anode and the cathode. This hydrophilic paper exchanges ions between the anode and the cathode for a short period of time. Eventually, it loses this functionality and allows for the exchange of liquids between the PEM^47^,^48^. Most MFC-powered studies have used chemical treatments, such as Nafion 117 solution^12^,^13^,^14^,^18^,^19^,^29^,^31^,^49^,^50^,^51^,^52^,^53^,^54^,^55^,^56^,^57^,^58^,^59^,^60^, polytetrafluoroethylene (PTFE)^12^,^37^,^51^,^52^,^53^,^56^,^61^, and sodium polystyrene sulfonate (Na-PSS)^31^, to allow protons to pass through a device efficiently. However, these chemically-treated PEMs are toxic and considerably increase the cost of the device compared to using parchment paper^62^. In particular, the use of Nafion showed superior battery performance, compared to other PEMs, but resulted in a lower conductivity at low humidity. There was also a significant volumetric size change with increasing humidity levels^29^. This makes it difficult to integrate these devices into paper-based bacterial metabolism-catalyzed battery^63^. Also, Nafion-based membranes are incompatible with microfabrication processes^38^. Some studies reported that a removal of PEM in air cathode MFC led to an increase in power production against the cost^63^,^64^. However, to the best of the authors’ knowledge, there has been no report of this finding in paper-based air cathode MFC system.

**Figure 1:**
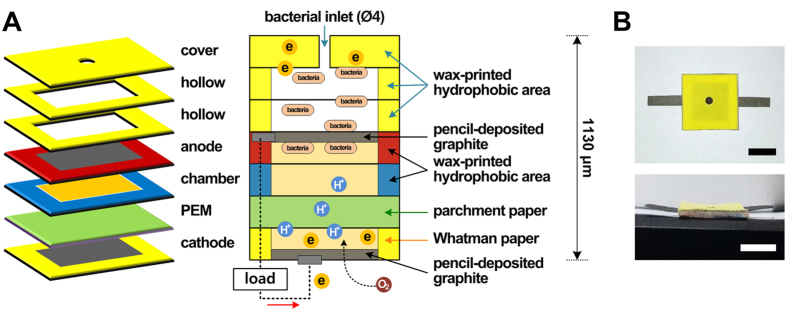
(**A**) Schematic diagram and (**B**) photo of the all-paper microbial-activated air cathode battery made with pencil-traced graphite electrodes. Scale bar = 15 mm. The low-cost, miniaturized, all-paper microbial electricity-generating device requires no additional chemical treatments.

**Figure 2:**
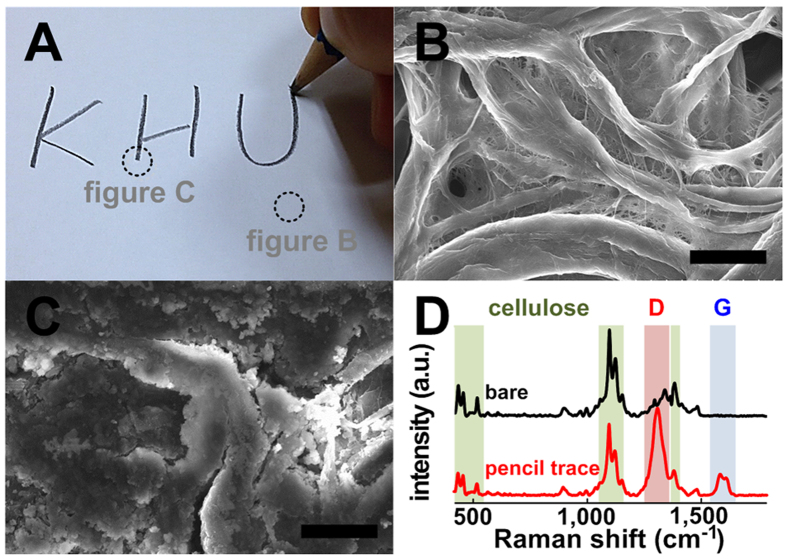
(**A**) Writing on paper with a pencil, (**B,C**) FE-SEM images, and (**D**) Raman spectra of the (**B**) bare paper and (**C**) 8B pencil-traced paper. Scale bar = 25 μm.

**Figure 3:**
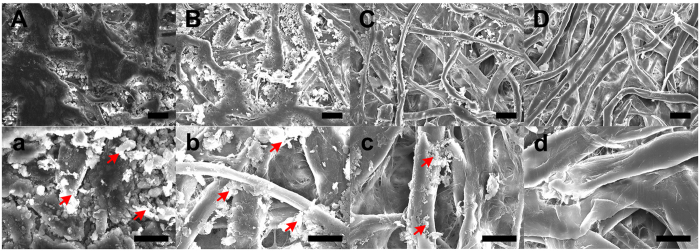
FE-SEM images of pencil-traced papers according to pencil hardness: (**Aa**) 8B, (**Bb**) 4B, (**Cc**) HB, and (**Dd**) 4H. Red arrows indicate the graphite particles deposited on the cellulose fiber in the paper. Scale bar = 50 μm for top panels. Scale bar = 25 μm for bottom panels.

**Figure 4:**
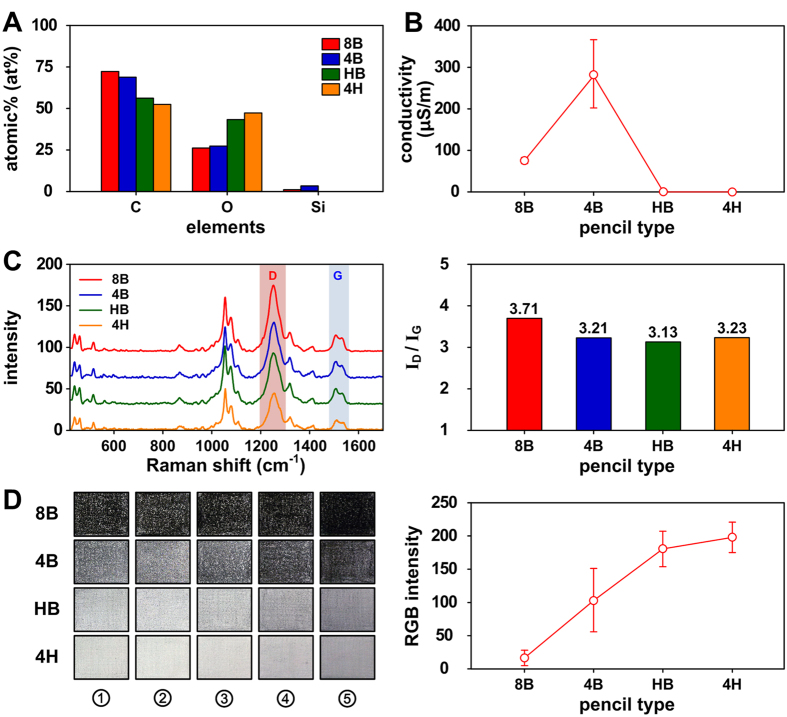
(**A**) Molecular properties, (**B**) electrical conductivity, (**C**) Raman spectra and I_D_/I_G_ ratios, and (**D**) reproducibility (four cycles) and RGB intensity of pencil-traced papers according to pencil hardness. ① – ③ electrodes were fabricated by normal tracing on paper (8 N), while ④ and ⑤ electrodes was fabricated by pressing-down tracing on paper (16 N).

**Figure 5:**
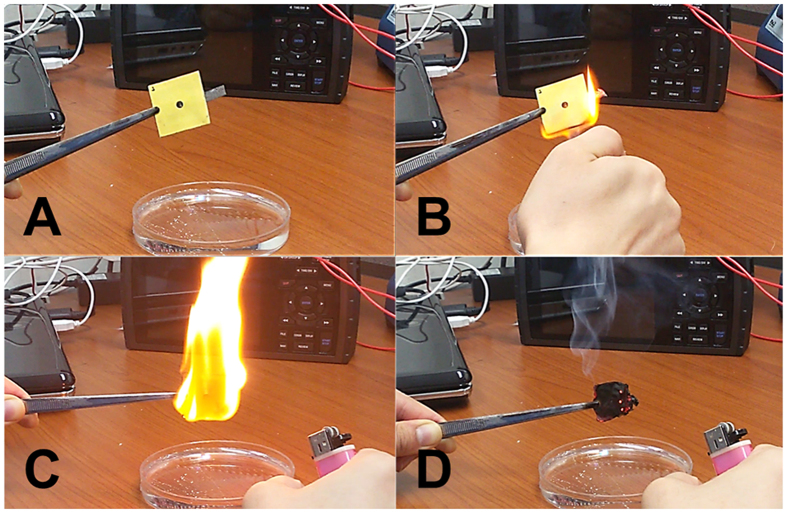
All-paper air cathode battery burned over 40 s (**A** to **D**).

An all-paper air cathode battery (Fig. 1B) is manually assembled with a glue stick by sandwiching the seven functional paper-based layers (Fig. S2). The size of a completely fabricated battery is about 30 × 30 × 1 mm^3^. The total material cost of the fabricated device was about 204 KRW ($0.20, Table S2). This all-paper air cathode battery could be cut or trimmed easily using scissors or a razor blade. Also, the bacteria-containing graphite and wax pattern on the burnable papers are disposable by incineration (Fig. 5). The paper-based battery was completely burned after 40 s. We believe that this capability is particularly useful in single-use medical power supplies that contact biological fluids or tissues^65^.

### Biopotential generation

**Figure 6:**
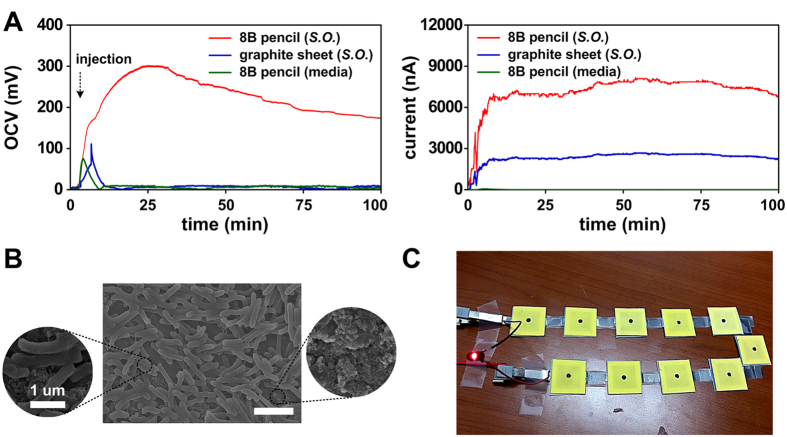
(**A**) Bacterial metabolism-catalyzed bioelectricity generation on pencil-traced air cathode paper batteries. *S.O.* indicates the wild-type *Shewanella oneidensis* MR-1 strain. (**B**) FE-SEM image of *Shewanella oneidensis* bacteria cells attached to the 8B pencil-traced anode. Scale bar = 2.5 μm. Dense bacterial cells attached onto graphite flakes deposited on the paper can be observed. (**C**) Operation of red light-emitting diode using a serially-connected microbial-activated air cathode battery paper platform as the power source.

Figure 6A shows the microbial-activated output voltage in the fully assembled air cathode battery paper with pencil-traced graphite electrodes. The proposed all-paper MFC showed a very fast start time to product the current (<10 s) after injection of the bacterial cells or media. This is due to the large surface area and higher liquid permeability of the paper substrate. After the injection of the bacteria-containing liquid, the open circuit voltage (OCV) continued to increase and reached a saturated voltage of 302.3 mV after 25 min, the mean OCV of our MFC was 250 ± 32 mV (n = 20). This pattern is consistent with the profile reported previously^12^. However, the output voltage of a single MFC was less than the OCV of a single conventional MFC (0.8 V)^66^. This is caused by O_2_ diffusion in the anode chamber^38^. Fortunately, the OCV of our single MFC is higher than those of paper-based air cathode MFCs reported previously (Table 1); these values were 230 mV for a *Shewanella oneidensis*-injected activated carbon-coated nickel cathode MFC with Nafion-treated PEM^12^ and 265 mV for a *Pseudomonas aeruginosa*-injected pencil-traced electrode (two anodes [530 mV] and one cathode) with a double bare paper-based PEM^30^. This might indicate that the cathode deposited with an 8B pencil, with dense graphite particles, is superior to the activated carbon-based cathode and 6B pencil-traced cathode. Our MFC sustained 67% of its peak OCV (about 200 mV) for 100 min after the *Shewanella oneidensis* was injected. After 100 min, the OCV and current levels decreased and were restored to near the baseline level within 100 min. Media-only injection in the same platform with an MFC with a graphite sheet cathode quickly showed a saturated OCV of about 100 mV; this was restored back near the baseline level within 10 min. The bioelectric current was calculated by measuring the microbial-activated voltage difference of a 100-kΩ load resistor. The media-only injected 8B pencil-traced MFC showed almost the baseline value (0 nA) after 100 min. Alternatively, the *Shewanella oneidensis*-injected 8B pencil-traced and graphite sheet MFCs showed currents of 7.5 μA and 2.5 μA; these were sustained without powering down for a period of 100 min. The current production for the 8B pencil-traced MFC was almost three times higher than that of the graphite sheet MFC. A maximum of 11 μA was observed in the *Shewanella oneidensis*-injected 8B pencil-traced single MFC. The current generation of our single MFC is higher than those of previously reported paper-based air cathode MFCs (Table 1), such as *Shewanella oneidensis*-injected activated carbon-coated nickel cathode MFCs with Nafion-treated PEMs (250 nA) and *Pseudomonas aeruginosa*-injected (2.5 μA) and *Shewanella putrefaciens*-injected (5 μA) pencil-traced MFCs with double bare paper-based PEMs. The principal mechanism of this bioelectric current is associated with the extracellular electron transfer (EET, Fig. 6B) in the MFC, which consisted of an anode chamber, PEM, cathode chamber, and an external conductive load (Fig. S9). The chemical energy created during this EET process was captured throughout the electron transport chain, Krebs cycle, and glycolysis^18^. The captured electrons are delivered through a load to the cathode. The protons generated via bacterial metabolism travel through the PEM towards the cathode. The electrons (*e*^−^) and protons (*H*^+^) combine to reduce the electron acceptor (oxygen, *O*_2_). Therefore, the redox couple is completed as:


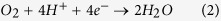


This electrochemical process produces the current in air cathode MFC devices. The mean and maximum power densities of our MFC were 2,270 nW (5.68 mW/m^2^) and 3,330 nW (8.33 mW/m^2^), respectively. This power density was higher than those reported previously: *Shewanella oneidensis*-injected activated carbon cathode MFCs (48 nW, 9.3 μW/m^2^) and *Pseudomonas aeruginosa*-injected (660 nW, 1.65 mW/m^2^) and *Shewanella putrefaciens*-injected (1,250 nW, 3.13 mW/m^2^) pencil-traced MFCs (Table 1). All biopotential generating experiments were primarily performed by single injection. Although multiple injections were acceptable, they showed relatively low performance compared to one time injection. The assembly of our MFCs (Fig. 1) was optimized to maximize the bioelectricity generation through various combinations of each layer. This indicates that our paper-based nano-graphitic pencil-traced air cathode battery is better at generating bacterial metabolism-catalyzed bioelectricity compared to previous air cathode paper battery devices. However, since the proposed paper-based MFC platforms showed low performance compared to conventional non-paper-based miniaturize MFC platforms (Table 1), the power/current density generated from paper-based MFC needs to be significantly improved for practical applications.

To evaluate the potential of our MFC platform in real applications, we serially-connected 10 MFCs to operate a light-emitting diode (>2V). After injection of the *Shewanella oneidensis*-containing liquid, we confirmed that ten of our MFCs (serially-connected) can drive a red light-emitting diode (Fig. 6C). This result suggests that the proposed solvent-free air cathode paper-based MFC devices can be used as environmental-friendly energy storages as well as in single-use medical power supplies that use organic matter.

## Conclusions

We developed a micro-fabricated, low cost, disposable, solvent-free air cathode all-paper MFC platform with pencil-traced graphite electrodes as a portable power source. The proposed battery platform, surrounded by wax-based hydrophobic barriers, consists of seven layers and includes an inlet reservoir, hollow areas, graphite-deposited hydrophilic anode and cathode, hydrophobic PEM, and hydrophilic bacteria-active chamber. Because the pencil with 8B hardness has high graphitic content, high electrical conductivity, high surface roughness of its nano-graphite, and the ability to make electrodes with high reproducibility, it was selected as the best electrode material for our all-paper air cathode MFC batteries. A fully integrated pencil-traced all-paper MFC (30 × 30 × 1 mm^3^) showed a very fast start-up time after injection of bacterial cells. A single micro-fabricated solvent-free air cathode all-paper MFC generated a current of 7,500 nA, an operating voltage of 302 mV, and a maximum power of 3,330 nW (8.33 mW/m^2^). The proposed solvent-free air cathode paper-based MFC device could be used as an environmentally-friendly power source for disposable diagnostic devices.

## Material and Methods

### Reagents and materials

Tryptic soy broth was purchased from Sigma Aldrich (St. Louis, MO, USA). All reagents were of analytical grade and all solutions were prepared using 18.3 MΩ·cm^−1^ distilled water. Wild-type *Shewanella oneidensis* MR-1 strain was purchased from ATCC (number 700550). Four types of pencils were purchased from STAEDTLER Mars GmbH & Co. KG (Nuernberg, Germany). A flexible graphite sheet (125-μm-thick and 99.8% pure) was purchased from GoodFellow (Coraopolis, PA, USA). Whatman cellulose chromatography paper (Grade 1, 180-μm-thick) was purchased from Sigma Aldrich. Parchment paper (<50-μm-thick) was purchased from Reynolds (Richmond, VA, USA) and 841 Super Shield^TM^ nickel conductive coating aerosol was purchased from MG chemical (Ontario, Canada).

### Instrumentation

The electrical potential between the anode and the cathode was measured using a data acquisition device (GL820, GRAPHTEC, Yokohama, Japan) with a 1 s interval. A load resistance of 100 kΩ was used to flow the current across the circuit. The resistances of the pencil-traced electrodes and Ni-coated paper were measured using a multi-meter (Fluke-115, Fluke, Everett, WA, USA).

The morphologies of the pencil traces on paper and the air cathode battery were investigated using an S-4700 field emission scanning electron microscope (FE-SEM; Hitachi, Tokyo, Japan) at an accelerating voltage of 20 kV. Energy dispersive X-ray spectroscopy (EDX; 7200-H, HORIBA, Northampton, England) and a SENTERRA confocal Raman system (Bruker Optics Inc., Billerica, Ma, USA) with a 785 nm diode laser source and a 20× objective lens were used to examine the elemental composition.

### Fabrication of air cathode paper-based battery

A paper-based air cathode battery consisting seven layers (Fig. 1) was fabricated with Whatman filter paper and parchment paper. Each layer primarily consisted of two parts: a 20 × 20 mm^2^ hydrophilic bacteria-active area and a hydrophobic barrier with a width of 5 mm (Fig. S1). The parchment paper was cut into 30 × 30 mm^2^ squares for the PEM layer. The other layers, which were designed by AutoCAD (Autodesk, San Rafael, CA, USA), were printed on 30 × 30 mm^2^ pieces of paper using a Xerox ColorQube 8570N printer (Fuji Xerox, Tokyo, Japan). Uniform impregnation of wax on the paper was performed in a drying oven at 130 °C for 45 s, after which the paper was dried at room temperature^67^. An inlet reservoir (cover layer) and the hydrophilic bacteria-active area (hollow layer) were cut using a 4-mm-diameter biopsy punch and a blade, respectively. The graphite was deposited by tracing a pencil on the hydrophilic areas of the anode and cathode layers. These were connected to wires made from nickel-coated paper (Fig. S3). The anode chamber layer and PEM layer were prepared without additional chemical treatments. Assembly of the seven prepared layers (Fig. S2) was completed using a glue stick (Fig. 1B).

### Battery operation

The two wires of the fabricated air cathode paper-based battery were connected to the data acquisition device at room temperature. It maintained the gap between the air cathode battery and floor to expose all air cathodes to the air. 300 μL of an anolyte containing wild-type *Shewanella Oneidensis* MR-1was introduced onto the inlet of the cover layer with a 1 mL syringe. The potentials of the open circuit and the closed circuit (with a resistance of 100 kΩ) were measured for a period of 100 min. The current was calculated with the resistor using Ohm’s law and the output power.

## Additional Information

**How to cite this article**: Lee, S. H. *et al*. A solvent-free microbial-activated air cathode battery paper platform made with pencil-traced graphite electrodes. *Sci. Rep.* **6**, 28588; doi: 10.1038/srep28588 (2016).

## Supplementary Material

Supplementary Information

## Figures and Tables

**Table 1:** Comparative study of bioelectricity generation using representative miniature MFCs.

Material	Electron accept	Electrode	PEM	Bacteria	OCV	Current	Power
paper	air-cathode	8B pencil (this study)	parchment	*S.O.* (300 μL)	302 mV	7500 nA	5.68 mW/m^2^
	air-cathode	6B pencil (two anodes)^30^	double bare	*S.P.* (400 μL)	250 mV	5000 nA	3.13 mW/m^2^
	air-cathode	6B pencil (two anodes)^30^	double bare	*P.A.* (400 μL)	265 mV	2500 nA	1.65 mW/m^2^
	air-cathode	graphite sheet (this study)	parchment	*S.O.* (300 μL)	100 mV	2500 nA	0.63 mW/m^2^
	air-cathode	AC-coated Ni & carbon ink (four-battery stack)^12^	Nafion	*S.O.* (25 μL)	230 mV	250 nA	9.30 μW/m^2^
plastic	air-cathode	PTFE-coated carbon cloth (double CEA)^68^	non	R.W. (6 mL)	315 mV	0.28 mA/m^2^	1460 mW/m^2^
	ferricyanide	reticulated vitreous carbon^49^	Nafion	*S.O.* (1.2 mL)	780 mV	44.4 mA/m^2^	22.2 mW/m^2^
	ferricyanide	graphite felt^49^	Nafion	*S.O.* (1.2 mL)	760 mV	20.5 mA/m^2^	9.8 mW/m^2^
	air-cathode	gold/carbon cloth (24-well array)^57^	Nafion	*S.O.* (650 μL)	N/A	6 mA/m^2^	2.69 mW/m^2^
	ferricyanide	gold/carbon cloth (24-well array)^58^	Nafion	*S.O.* (650 μL)	538 mV	5.54 mA/m^2^	0.4 mW/m^2^
	ferricyanide	chromium-coated gold^59^	Nafion	*S.C.* (16 μL)	450 mV	150 mA/m^2^	0.23 mW/m^2^
	fumarate	carbon cloth^60^	Nafion	*G.S.* (N/A)	N/A	3147 mA/m^2^	N/A
	fumarate	gold^60^	Nafion	*G.S.* (N/A)	N/A	688 mA/m^2^	N/A

*AC, activated carbon. PTFE, polytetrafluoroethylene. CEA, cloth electrode assembly. *S.O., Shewanella oneidensis. S.P., Shewanella putrefaciens. P.A., Pseudomonas aeruginosa.* R.W., real wastewater obtained from Corvallis Wastewater Treatment Plant (Corvallis, OR). *G.S.*, *Geobacter sulfurreducens*. *S.C.*, *Saccharomyces cerevisiae*.

## References

[b1] Wu X. E., Guo Y. Z., Chen M. Y. & Chen X. D. Fabrication of flexible and disposable enzymatic biofuel cells. Electrochim. Acta 98, 20–24 (2013).

[b2] Zhang L. . Small-size biofuel cell on paper. Biosens. Bioelectron. 35, 155–159 (2012). 22417872 10.1016/j.bios.2012.02.035

[b3] Zebda A. . Mediatorless high-power glucose biofuel cells based on compressed carbon nanotube-enzyme electrodes. Nat. Commun. 2, 370 (2011). 21712818 10.1038/ncomms1365 PMC3156815

[b4] Barton S. C., Gallaway J. & Atanassov P. Enzymatic biofuel cells for implantable and microscale devices. Chem. Rev. 104, 4867–4886 (2004). 15669171 10.1021/cr020719k

[b5] Yan Y., Zheng W., Su L. & Mao L. Carbon-Nanotube-Based Glucose/O2 Biofuel Cells. Adv. Mater. 18, 2639–2643 (2006).

[b6] Kim J., Jia H. & Wang P. Challenges in biocatalysis for enzyme-based biofuel cells. Biotechnol. Adv. 24, 296–308 (2006). 16403612 10.1016/j.biotechadv.2005.11.006

[b7] Liu C., Alwarappan S., Chen Z., Kong X. & Li C.-Z. Membraneless enzymatic biofuel cells based on graphene nanosheets. Biosens. Bioelectron. 25, 1829–1833 (2010). 20056403 10.1016/j.bios.2009.12.012

[b8] Shitanda I., Kato S., Hoshi Y., Itagaki M. & Tsujimura S. Flexible and high-performance paper-based biofuel cells using printed porous carbon electrodes. Chem. Commun. (Camb). 49, 11110–11112 (2013). 24146105 10.1039/c3cc46644b

[b9] Taher H., Al-Zuhair S., Al-Marzouqi A. H., Haik Y. & Farid M. M. A review of enzymatic transesterification of microalgal oil-based biodiesel using supercritical technology. Enzyme Res. 2011, 468292 (2011). 21915372 10.4061/2011/468292 PMC3170906

[b10] Rahman M. M., Ahammad A. J. S., Jin J.-H., Ahn S. J. & Lee J.-J. A Comprehensive Review of Glucose Biosensors Based on Nanostructured Metal-Oxides. Sensors 10, 4855–4886 (2010). 22399911 10.3390/s100504855 PMC3292151

[b11] Nguyen T. H., Fraiwan A. & Choi S. Paper-based batteries: A review. Biosens. Bioelectron. 54, 640–649 (2014). 24333937 10.1016/j.bios.2013.11.007

[b12] Lee H. & Choi S. An origami paper-based bacteria-powered battery. Nano Energy 15, 549–557 (2015).

[b13] Fraiwan A. . Microbial Power-Generating Capabilities on Micro-/Nano- structured Anodes in Micro-sized Microbial Fuel Cells. Fuel Cells 14, 801–809 (2014).

[b14] Wang H. Y., Bernarda A., Huang C. Y., Lee D. J. & Chang J. S. Micro-sized microbial fuel cell: A mini-review. Bioresour. Technol. 102, 235–243 (2011). 20709539 10.1016/j.biortech.2010.07.007

[b15] Qian F. & Morse D. E. Miniaturizing microbial fuel cells. Trends Biotechnol. 29, 62–69 (2011). 21075467 10.1016/j.tibtech.2010.10.003

[b16] Winfield J., Chambers L. D., Rossiter J., Greenman J. & Ieropoulos I. Urine-activated origami microbial fuel cells to signal proof of life. J. Mater. Chem. A 3, 7058–7065 (2015).

[b17] Choi S. Microscale microbial fuel cells: Advances and challenges. Biosens. Bioelectron. 69, 8–25 (2015). 25703724 10.1016/j.bios.2015.02.021

[b18] Fraiwan A., Lee H. & Choi S. A Multi-Anode Paper-based Microbial Fuel Cell :A Potential Power Source for Disposable Biosensors. IEEE Sens. J. 14, 3385–3390 (2014).

[b19] Mukherjee S. . A microliter-scale microbial fuel cell array for bacterial electrogenic screening. Sensors Actuators, A Phys. 201, 532–537 (2013).

[b20] Rittmann B. E. Opportunities for renewable bioenergy using microorganisms. Biotechnol. Bioeng. 100, 203–212 (2008). 18431744 10.1002/bit.21875

[b21] Logan B. E. & Regan J. M. Electricity-producing bacterial communities in microbial fuel cells. Trends Microbiol. 14, 512–518 (2006). 17049240 10.1016/j.tim.2006.10.003

[b22] Logan B. E. Scaling up microbial fuel cells and other bioelectrochemical systems. Appl. Microbiol. Biotechnol. 85, 1665–1671 (2010). 20013119 10.1007/s00253-009-2378-9

[b23] Liu H., Xiang Y., Lu Y. & Crooks R. M. Aptamer-based origami paper analytical device for electrochemical detection of adenosine. Angew. Chemie Int. Ed. 51, 6925–6928 (2012). 10.1002/anie.201202929 PMC3486962 22639438

[b24] Li W. . Battery-triggered ultrasensitive electrochemiluminescence detection on microfluidic paper-based immunodevice based on dual-signal amplification strategy. Anal. Chim. Acta 767, 66–74 (2013). 23452788 10.1016/j.aca.2012.12.053

[b25] Wang S. . Battery-triggered microfluidic paper-based multiplex electrochemiluminescence immunodevice based on potential-resolution strategy. Lab Chip 12, 4489–4498 (2012). 22971643 10.1039/c2lc40707h

[b26] Yetisen A. K., Akram M. S. & Lowe C. R. Paper-based microfluidic point-of-care diagnostic devices. Lab Chip 13, 2210–22151 (2013). 23652632 10.1039/c3lc50169h

[b27] Metters J. P., Houssein S. M., Kampouris D. K. & Banks C. E. Paper-based electroanalytical sensing platforms. Anal. Methods 5, 103–110 (2013).

[b28] Nyholm L., Nyström G., Mihranyan A. & Strømme M. Toward flexible polymer and paper-based energy storage devices. Adv. Mater. 23, 3751–3769 (2011). 21739488 10.1002/adma.201004134

[b29] Fraiwan A. & Choi S. Bacteria-powered battery on paper. Phys. Chem. Chem. Phys. 16, 26288–26293 (2014). 25363848 10.1039/c4cp04804k

[b30] Veerubhotla R., Bandopadhyay A., Das D. & Chakraborty S. Instant power generation from an air-breathing paper and pencil based bacterial bio-fuel cell. Lab Chip 15, 2580–2583 (2015). 25998260 10.1039/c5lc00211g

[b31] Fraiwan A., Mukherjee S., Sundermier S., Lee H. S. & Choi S. A paper-based microbial fuel cell: Instant battery for disposable diagnostic devices. Biosens. Bioelectron. 49, 410–414 (2013). 23807233 10.1016/j.bios.2013.06.001

[b32] Esquivel J. P. . Fuel cell-powered microfluidic platform for lab-on-a-chip applications. Lab Chip 12, 74–79 (2012). 22072241 10.1039/c1lc20426b

[b33] Esquivel J. P., Del Campo F. J., Gómez de la Fuente J. L., Rojas S. & Sabaté N. Microfluidic fuel cells on paper: meeting the power needs of next generation lateral flow devices. Energy Environ. Sci. 7, 1744 (2014).

[b34] Choi G., Hassett D. J. & Choi S. A paper-based microbial fuel cell array for rapid and high-throughput screening of electricity-producing bacteria. Analyst 140, 4277–4283 (2015). 25939879 10.1039/c5an00492f

[b35] Oyola-Reynoso S. . Draw your assay: Fabrication of low-cost paper-based diagnostic and multi-well test zones by drawing on a paper. Talanta 144, 289–293 (2015). 26452824 10.1016/j.talanta.2015.06.018

[b36] Gierlinger N., Keplinger T. & Harrington M. Imaging of plant cell walls by confocal Raman microscopy. Nat. Protoc. 7, 1694–1708 (2012). 22918387 10.1038/nprot.2012.092

[b37] Qian W. . Human hair-derived carbon flakes for electrochemical supercapacitors. Energy Environ. Sci. 7, 379–386 (2013).

[b38] Choi S. . A μL-scale micromachined microbial fuel cell having high power density. Lab Chip 11, 1110–1117 (2011). 21311808 10.1039/c0lc00494d

[b39] Kurra N. & Kulkarni G. U. Pencil-on-paper: electronic devices. Lab Chip 13, 2866–2873 (2013). 23753048 10.1039/c3lc50406a

[b40] Lin C.-W., Zhao Z., Kim J. & Huang J. Pencil drawn strain gauges and chemiresistors on paper. Sci. Rep. 4, 3812 (2014). 24448478 10.1038/srep03812 PMC3898045

[b41] Pimenta M. A. . Studying disorder in graphite-based systems by Raman spectroscopy. Phys. Chem. Chem. Phys. 9, 1276–1291 (2007). 17347700 10.1039/b613962k

[b42] Caņado L. G. . General equation for the determination of the crystallite size La of nanographite by Raman spectroscopy. Appl. Phys. Lett. 88, 163106 (2006).

[b43] Mirica K. a., Weis J. G., Schnorr J. M., Esser B. & Swager T. M. Mechanical Drawing of Gas Sensors on Paper. Angew. Chemie Int. Ed. 51, 10740–10745 (2012). 10.1002/anie.201206069 PMC3494289 23037938

[b44] Glavan A. C. . Rapid fabrication of pressure-driven open-channel microfluidic devices in omniphobic RF paper. Lab Chip 13, 2922–2930 (2013). 23719764 10.1039/c3lc50371b

[b45] Renault C., Anderson M. J. & Crooks R. M. Electrochemistry in hollow-channel paper analytical devices. J. Am. Chem. Soc. 136, 4616–4623 (2014). 24635569 10.1021/ja4118544

[b46] Renault C., Li X., Fosdick S. E. & Crooks R. M. Hollow-channel paper analytical devices. Anal. Chem. 85, 7976–7979 (2013). 23931456 10.1021/ac401786h

[b47] Lan W. . Paper-Based Potentiometric Ion Sensing. Anal. Chem. 86, 9548–9553 (2014). 25197763 10.1021/ac5018088

[b48] Hu J. . All-Solid-State Reference Electrodes Based on Colloid-Imprinted Mesoporous Carbon and Their Application in Disposable Paper-based Potentiometric Sensing Devices. Anal. Chem. 87, 2981–2987 (2015). 25630744 10.1021/ac504556s

[b49] Ringeisen B. R. . High power density from a miniature microbial fuel cell using Shewanella oneidensis DSP10. Environ. Sci. Technol. 40, 2629–2634 (2006). 16683602 10.1021/es052254w

[b50] Taghavi M. . Self sufficient wireless transmitter powered by foot-pumped urine operating wearable MFC. Bioinspir. Biomim. 11, 016001 (2015). 26657063 10.1088/1748-3190/11/1/016001

[b51] Cheng S., Liu H. & Logan B. E. Power densities using different cathode catalysts (Pt and CoTMPP) and polymer binders (nafion and PTFE) in single chamber microbial fuel cells. Environ. Sci. Technol. 40, 364–369 (2006). 16433373

[b52] Wei B., Tokash J. C., Chen G., Hickner M. A. & Logan B. E. Development and evaluation of carbon and binder loading in low-cost activated carbon cathodes for air-cathode microbial fuel cells. RSC Adv. 12751–12758 10.1039/c2ra21572a (2012).

[b53] Zhang F. . Mesh optimization for microbial fuel cell cathodes constructed around stainless steel mesh current collectors. J. Power Sources 196, 1097–1102 (2011).

[b54] Zhang X., Xia X., Ivanov I., Huang X. & Logan B. E. Enhanced activated carbon cathode performance for microbial fuel cell by blending carbon black. Environ. Sci. Technol. 48, 2075–2081 (2014). 24422458 10.1021/es405029y

[b55] Oliveira V. B., Simões M., Melo L. F. & Pinto A. M. F. R. Overview on the developments of microbial fuel cells. Biochem. Eng. J. 73, 53–64 (2013).

[b56] Watson V. J., Nieto Delgado C. & Logan B. E. Influence of chemical and physical properties of activated carbon powders on oxygen reduction and microbial fuel cell performance. Environ. Sci. Technol. 47, 6704–6710 (2013). 23692057 10.1021/es401722j

[b57] Hou H., Li L., de Figueiredo P. & Han A. Air-cathode microbial fuel cell array: A device for identifying and characterizing electrochemically active microbes. Biosens. Bioelectron. 26, 2680–2684 (2011). 20655725 10.1016/j.bios.2010.06.037

[b58] Hou H., Li L., Cho Y., de Figueiredo P. & Han A. Microfabricated microbial fuel cell arrays reveal electrochemically active microbes. PLoS One 4, 1–8 (2009). 10.1371/journal.pone.0006570 PMC2718701 19668333

[b59] Chiao M., Lam K. B. & Lin L. Micromachined microbial and photosynthetic fuel cells. J. Micromechanics Microengineering 16, 2547–2553 (2006).

[b60] Richter H. . Electricity Generation by Geobacter sulfurreducensAttached to Gold Electrodes. Langmuir 24, 4376–4379 (2008). 18303924 10.1021/la703469y

[b61] Desmaële D., Renaud L. & Tingry S. A wireless sensor powered by a flexible stack of membraneless enzymatic biofuel cells. Sensors Actuators, B Chem. 220, 583–589 (2015).

[b62] Lee Y.-Y., Kim T. G. & Cho K.-S. Effects of proton exchange membrane on the performance and microbial community composition of air-cathode microbial fuel cells. J. Biotechnol. 211, 130–137 (2015). 26235818 10.1016/j.jbiotec.2015.07.018

[b63] Li W.-W., Sheng G.-P., Liu X.-W. & Yu H.-Q. Recent advances in the separators for microbial fuel cells. Bioresour. Technol. 102, 244–252 (2011). 20382524 10.1016/j.biortech.2010.03.090

[b64] Liu H. & Logan B. E. Electricity generation using an air-cathode single chamber microbial fuel cell in the presence and absence of a proton exchange membrane. Environ. Sci. Technol. 38, 4040–4046 (2004). 15298217 10.1021/es0499344

[b65] Siegel A. C. . Foldable printed circuit boards on paper substrates. Adv. Funct. Mater. 20, 28–35 (2010).

[b66] Oh S.-E. & Logan B. E. Voltage reversal during microbial fuel cell stack operation. J. Power Sources 167, 11–17 (2007).

[b67] Choi S., Kim S.-K., Lee G.-J. & Park H.-K. Paper-based 3D microfluidic device for multiple bioassays. Sensors Actuators B Chem. 219, 245–250 (2015).

[b68] Fan Y., Hu H. & Liu H. Enhanced Coulombic efficiency and power density of air-cathode microbial fuel cells with an improved cell configuration. J. Power Sources 171, 348–354 (2007).

