# Supplementary material for: A solvent-free microbial-activated air cathode battery paper platform made with pencil-traced graphite electrodes
Source: Sci Rep. 2016 Jun 23;6:28588. doi: 10.1038/srep28588 (PMC4917852; doi:10.1038/srep28588)
Supplement: Supplementary Information [file srep28588-s1.pdf]

## Supplementary Information

# A solvent-free microbial-activated air cathode battery paper platform made with pencil-traced graphite electrodes

Seung Ho Lee, Ju Yeon Ban, Chung-Hun Oh, Hun-Kuk Park*, Samjin Choi*

Department of Biomedical Engineering, College of Medicine, Kyung Hee University, Seoul 130-701, Korea

***Address for correspondence:**

Samjin Choi, Ph.D.,

Department of Biomedical Engineering,

College of Medicine,

Kyung Hee University,

1 Hoegi-dong, Dongdaemun-gu,

Seoul 130-701, Korea

Tel: +82 2 961 0290

Fax: +82 2 6008 5535

E-mail: medchoi@khu.ac.kr

Figures: 9

Table: 2

## ■ Table

**Table S1. Peak assignment of SERS spectra for cellulose.**[1–3]

| peak ($cm^{-1}$) | assignment |
|---|---|
| 437, 458, 520 | C–C–C, C–O–C, O–C–C, and O–C–O skeletal bending |
| 1095 | C–N stretching in proteins, chain C–C stretching in proteins, and C–O stretching in carbohydrates |
| 1120 | C–O–C symmetric stretching |
| 1337 | H–C–H (wagging), H–C–C, H–O–C, and C–O–H (rocking) bending |
| 1380 | ring C–C stretching |

**Table S2. Cost of materials for the fabrication of all-paper air cathode batteries on the laboratory-scale.***

| material | required amount | cost (KRW) | cost ($) |
|---|---|---|---|
| Whatman chromatography paper (200×200 $mm^2$) | 60×95 $mm^2$ | 136 | 0.12 |
| wax printing | 30×90 $mm^2$ | 18 | 0.02 |
| 8B pencil trace | four strokes | 0.4 | 0.01 |
| parchment paper (PEM) | 30×30 $mm^2$ | 12 | 0.01 |
| Ni spray | 1.3 mL | 37 | 0.03 |
| glue stick | 20 mg | 0.7 | 0.01 |
| sum | | 204.1 | 0.20 |

*Exchange rate for $ to KRW, 1 USD=1138 KRW.

## ■ Figures

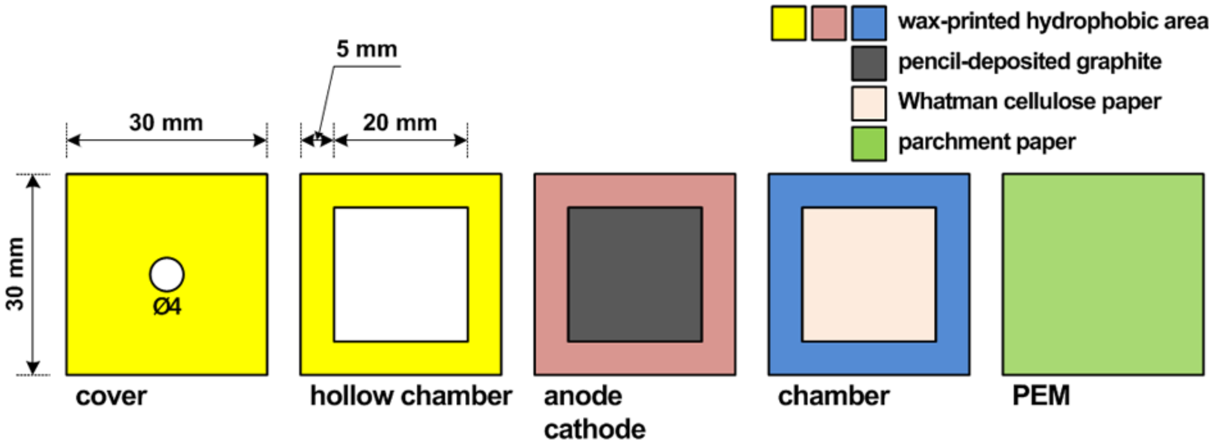


**Figure S1. Design concept of all-paper microbial-activated air cathode battery.**

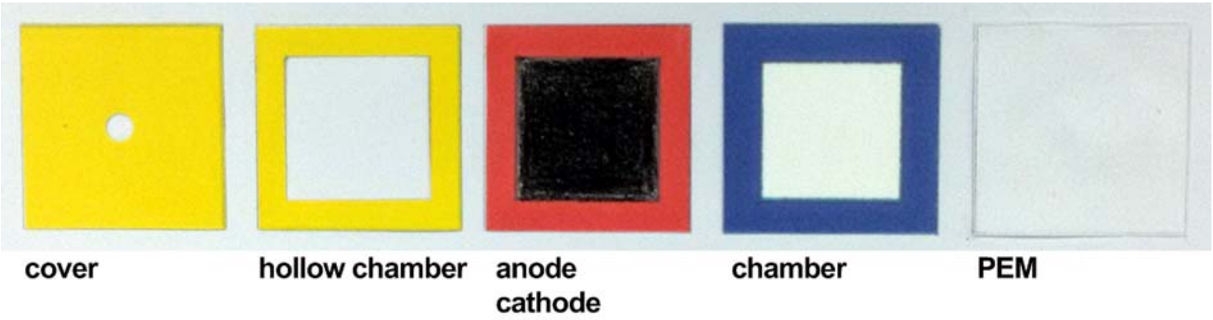


**Figure S2. Photos of each layer for all-paper microbial-activated air cathode battery.**

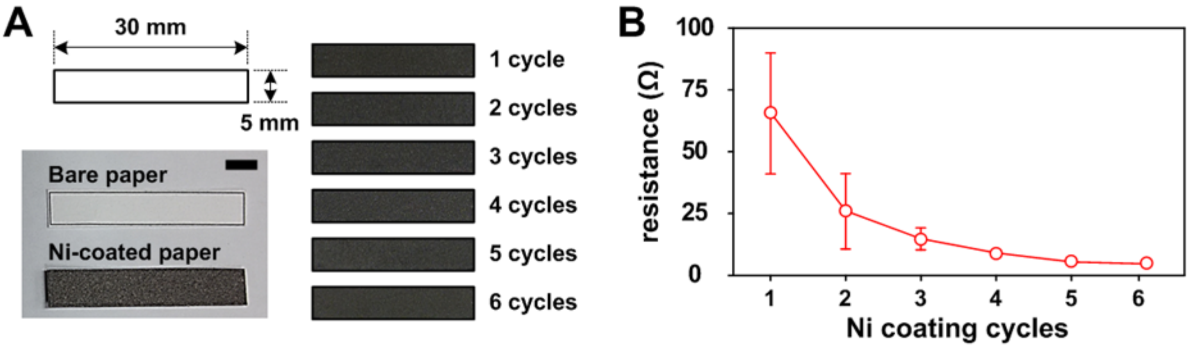


**Figure S3. (A) Photo and (B) electrical properties of paper-based Ni-coated wires according to the number of coating cycles.** Scale bar=5 mm.

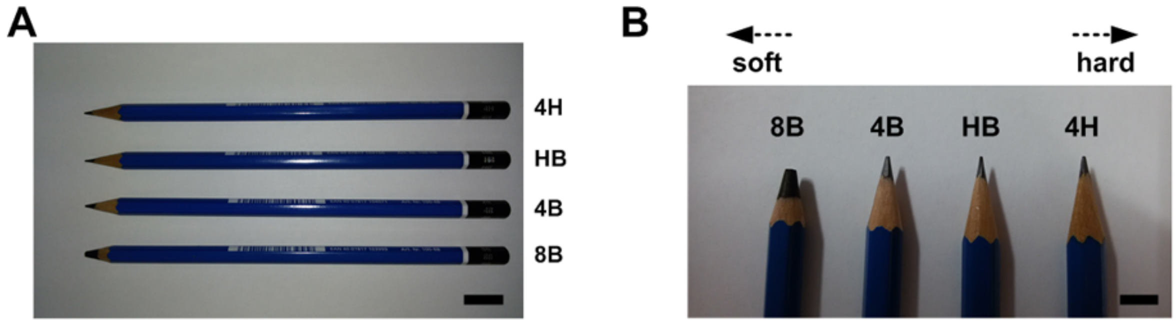


**Figure S4. Four types of pencils with different hardnesses (STAEDTLER Mars GmbH & Co. KG, Nuernberg, Germany).** (A) Scale bar=10 mm. (B) Scale bar=8 mm.

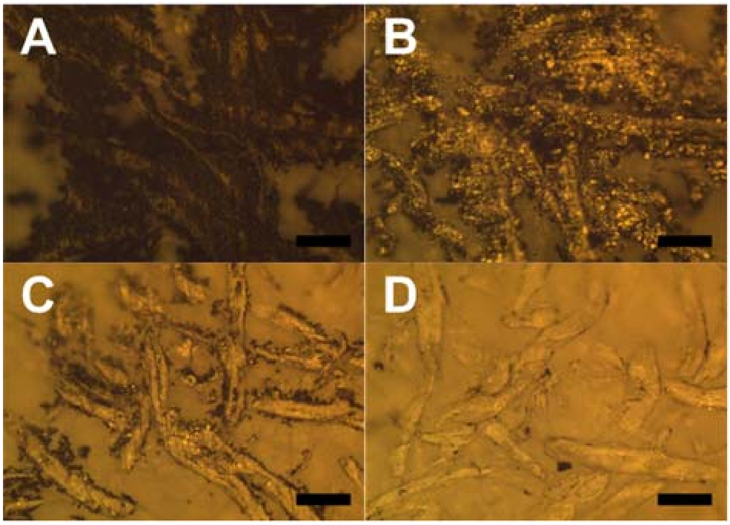


**Figure S5. Optical microscopy images of pencil-traced paper according to pencil hardness: (A) 8B, (B) 4B, (C) HB, and (D) 4H.** Scale bar=50 μm.

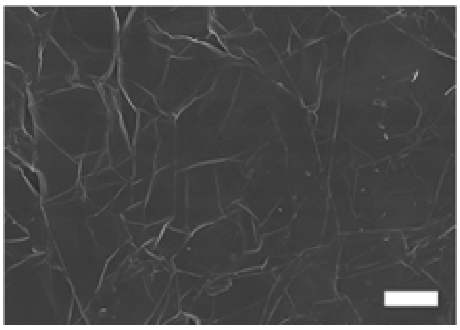

**Figure S6. FE-SEM image of graphite sheet.** Scale bar=5 μm. The graphite sheet showed a smooth surface with fewer defects compared to the pencil-traced paper substrates (Fig. 3).

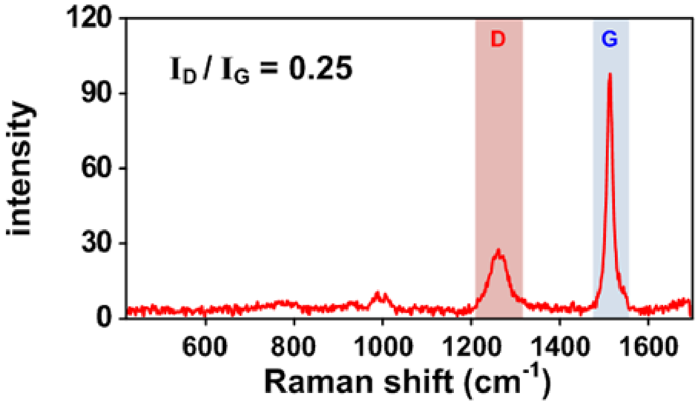


**Figure S7. Raman spectrum and $I_D/I_G$ ratio of graphite sheet.**

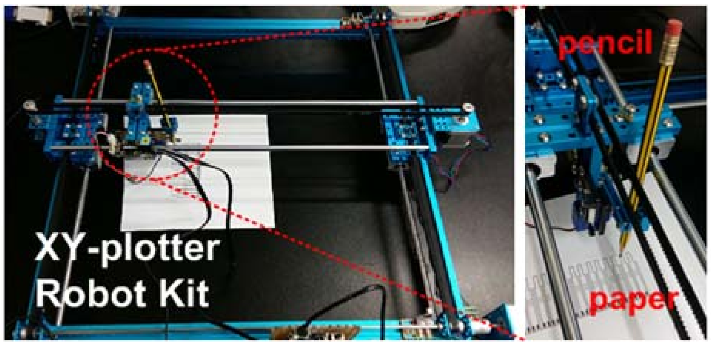


**Figure S8. Fabrication of pencil-traced electrodes on paper using an XY-plotter robot kit to achieve higher reproducibility (future study).**

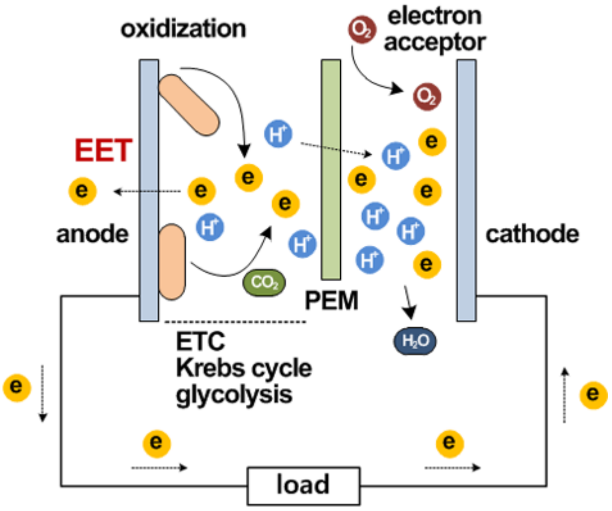


**Figure S9. Schematic diagram of bioelectricity generation for paper-based air cathode MFC.** EET, extracellular electron transfer. ETC, electron transport chain.

## References


1. Gierlinger, N., Keplinger, T. & Harrington, M. Imaging of plant cell walls by confocal Raman microscopy. *Nat. Protoc.* **7,** 1694–1708; doi:10.1038/nprot.2012.092 (2012).

2. Szymańska-Chargot, M., Cybulska, J. & Zdunek, A. Sensing the structural differences in cellulose from apple and bacterial cell wall materials by Raman and FT-IR Spectroscopy. *Sensors* **11,** 5543–5560; doi:10.3390/s110605543 (2011).

3. Wiley, J. H. & Atalla, R. H. Band Assignments in the Raman Spectra of Celluloses. *Carbohydr. Res.* **160,** 113–129 (1987).
